# Vaccine-Induced Carbohydrate-Specific Memory B Cells Reactivate During Rodent Malaria Infection

**DOI:** 10.3389/fimmu.2019.01840

**Published:** 2019-08-09

**Authors:** Hayley Joseph, Qiao Ye Tan, Ramin Mazhari, Emily M. Eriksson, Louis Schofield

**Affiliations:** ^1^Division of Population Health and Immunity, Walter and Eliza Hall Institute of Medical Research, Melbourne, VIC, Australia; ^2^Department of Medical Biology, The University of Melbourne, Melbourne, VIC, Australia; ^3^Australian Institute of Tropical Health and Medicine, James Cook University, Townsville, QLD, Australia

**Keywords:** malaria, B cell immunity, vaccines, memory failure, glycosylphosphatidylinositol (GPI)

## Abstract

A long-standing challenge in malaria is the limited understanding of B cell immunity, previously hampered by lack of tools to phenotype rare antigen-specific cells. Our aim was to develop a method for identifying carbohydrate-specific B cells within lymphocyte populations and to determine whether a candidate vaccine generated functional memory B cells (MBCs) that reactivated upon challenge with *Plasmodium* (pRBCs). To this end, a new flow cytometric probe was validated and used to determine the kinetics of B cell activation against the candidate vaccine glycosylphosphatidylinositol conjugated to Keyhole Limpet Haemocyanin (GPI-KLH). Additionally, immunized C57BL/6 mice were rested (10 weeks) and challenged with pRBCs or GPI-KLH to assess memory B cell recall against foreign antigen. We found that GPI-specific B cells were detectable in GPI-KLH vaccinated mice, but not in *Plasmodium*-infected mice. Additionally, in previously vaccinated mice GPI-specific IgG1 MBCs were reactivated against both pRBCs and synthetic GPI-KLH, which resulted in increased serum levels of anti-GPI IgG in both challenge approaches. Collectively our findings contribute to the understanding of B cell immunity in malaria and have important clinical implications for inclusion of carbohydrate conjugates in malaria vaccines.

## Introduction

The key criteria for long term immunity stems from the production of functional memory cells that are capable of reactivating, and boosting, in response to antigenic challenge. Both HIV and malaria are examples whereby protective natural immunity does not occur and there is accumulating evidence of ineffectual or atypical memory B cells (MBCs), although their exact function in malaria is still unknown (1–3). Furthermore, *Plasmodium sp*. have evolved mechanisms to evade the immune system, such as allelic diversity and antigenic variation. To date there are three lines of evidence to support the theory for B cell failure in malaria: (1) the observed partial acquisition of humoral immunity during natural exposure (4), which only persists during constant exposure, (2) the presence of atypical MBCs (4), and (3) the unexpected failure of the licensed RTS,S vaccine to induce long-term protection (5).

The only licensed malaria vaccine, RTS,S, specifically targets the circumsporozoite protein (CSP), which is expressed on the surface of the sporozoite prior to hepatocyte invasion (6), and not during blood-stage infection. Despite initial promising field trials, larger scale administration has resulted in a significant public health issue, since vaccine efficacy has been reported to be ~30%, protection wanes quickly and antibody levels are not maintained (5, 7). These observations are suggestive of B cell memory failure, yet there is a lack of cellular evidence to substantiate this possibly because of earlier methodological constraints to directly study antigen-specific B cells, without the need for pre-enrichment steps.

Recent application of tetramer probes in flow cytometry have given insight into *Plasmodium*-specific B cell immunology (1, 8, 9), but knowledge surrounding B cell immunity in malaria is still lagging behind other cellular immunology. In malaria, tetramers have been used to either enrich for antigen-specific cells (via anti-PE magnetic beads) (1), or in master mixes containing other cell surface markers (9). These previous studies target protein antigen, whereas our antigen of interest was a small 9 k-Da carbohydrate. Therefore, we aimed to design an alternative two-step staining flow cytometry protocol to identify these carbohydrate-specific B cells.

Another identified challenge in malaria is if the efficacy of the RTS,S can be improved with potential inclusion of other antigenic targets, such as glycosylphosphatidylinositol (GPI). GPI is expressed freely or functions as a membrane anchor for numerous malaria proteins important for essential parasite processes including facilitating red blood cell invasion (10). Additionally GPI anchors CSP to the surface of the parasite, the target protein in RTS,S. As a free moiety, GPI acts as a toxin similar to LPS (11–14) and previous human (15, 16) and murine (11) studies have demonstrated the importance of anti-GPI antibodies in protection against severe disease or fatality. GPI is structurally conserved across strains (17), species (18–21), and expressed during all lifecycle stages, making it a favorable target.

We therefore sought in this study to address two questions. The first to determine if we could develop a GPI specific probe and, by doing so, track the kinetic response of B cells to a novel carbohydrate-based vaccine (GPI) against *Plasmodium*. The second was to assess if we could detect reactivation of GPI-specific MBCs in GPI-vaccinated mice or *Plasmodium*-infected mice in response to synthetic GPI or native GPI (blood-stage infection). Our findings contribute to understanding the role B cells play in clinical immunity.

## Materials and Methods

### Vaccine Constructs

Synthetic glycosylphosphatidylinositol (GPI) was conjugated to maleimide activated Keyhole Limpet Haemocyanin (KLH) using 2-iminothiolane and stored at −80°C until use (GPI-KLH). Four-hydroxy-3-nitrophenyl acetyl-Osu (NP-Osu) (Biosearch Technologies, USA) was conjugated to KLH (Sigma-Aldrich, USA; molar ratio between 13 and 20) according to manufacturer's instructions and stored at −20°C until use (NP-KLH).

### Mice: Infections and Immunizations

For all experiments 6- to 8-week old inbred male and female C57BL/6 mice were used and all procedures complied with the Walter and Eliza Hall Institute Animal Ethics Committee requirements. Infections and immunizations are outlined in Supplementary Table 1. Mice were immunized with NP-KLH, GPI-KLH, or KLH precipitated on 10% alum (Sigma-Aldrich, USA) (Supplementary Table 1). Mice that were infected with *Plasmodium berghei* by intraperitoneal (*i.p.)* injection were given different numbers of parasitized red blood cells (pRBCs) depending on the experiment (Supplementary Table 1) and referred to as the group “Plasmodium.” All experimental mice were drug-cured (Supplementary Table 1) (22).

Mice included in memory recall experiments, were rested a minimum of 10 weeks and then injected (“challenged”) with GPI-KLH *i.p*. or pRBCs *i.p*. to assess reactivation of MBCs (Supplementary Table 1). A subset of GPI-KLH vaccinated mice were included that were not injected at 10 weeks to represent a baseline control. No more than 1 week prior to injection with GPI-KLH or pRBCs, sera was collected via mandible bleeds. Experiments were independently repeated 3 times and data pooled for statistical analysis.

### Preparation of Antigen-Specific Probes for Flow Cytometry

The GPI probe was constructed by conjugating GPI to maleimide activated bovine serum albumin (BSA) (ThermoScientific, USA) and biotinylating with Biotin (BIO)-XX (Invitrogen, USA) according to manufacturer's instructions (ratio of 1:1). The label is referred to as GPI-BSA-BIO. To determine optimum ratio of biotin to Streptavidin-phycoerythrin (Strep-PE) (BD Pharmingen, USA) for two-step staining in flow cytometry, titrations were carried out on splenocytes from previously vaccinated mice. To exclude background staining of BSA, control reagents of BSA-BIO were prepared as above and used in separate staining wells. To detect NP-specific B cells, NIP-CAP-Osu (succinimide ester of 3-nitro-4-hydroxy-5-iodophenylacetic acid spaced with caproic acid) (Biosearch Technologies, USA) was conjugated to PE (20 μg: 1 mg) (Life Technologies, USA).

### Flow Cytometry

Single cell suspensions from spleen or bone marrow were prepared and 3 × 10^6^ cells were added per well for staining. For detection of GPI-specific B cells, a two-step staining method was utilized. Gating strategies are included as supplementary data (Supplementary Figure 1). The primary master-mix stained for either GPI-BSA-BIO or background staining of BSA-BIO. There was no detectable non-specific staining with BSA-BIO (see Supplementary Figure 2). Following incubation, splenocytes were washed and re-suspended in secondary mastermixes. The following fluorochrome-conjugated monoclonal antibodies were used to stain B cells in the secondary mastermix: Pacific Blue (PB) anti-CD19 (clone 1D3, Ebioscience, USA), allophycocyanin (APC) anti-Immunoglobulin G1 (IgG1) (clone X56), PECy7 anti-CD95 (clone Jo2), fluorescein isothiocyanate (FITC), anti-CD38 (clone Ab90). IgD+ and/or Gr1+ cells were determined by Alexa 680 (A680) anti-IgD (clone 1126C) and A680 anti-Gr1 (clone 8C5) and excluded using a dump channel. This was to exclude naïve IgD positive B cells and granulocytes. For plasma cells, there was a one-step staining process with the mastermix containing PECy7 anti-B220 (clone RA3-6B2), and PE anti-CD138 (clone 281-2) (BD biosciences, USA).

For all staining, dead cells were excluded using live/dead fixable yellow dead cell staining kits (Life technologies, USA). Antigen specificity was determined using GPI-BSA-BIO/Strep-PE or NP-PE. Fluorescence Minus One (FMO) controls were included to allow accurate gating. Sample analysis was performed on a FortessaX20 flow cytometer and Cell Quest software packages (BD biosciences, USA). A minimum of 500 000 events was acquired. Data were analyzed using FlowJo Version 9.9.6 software (TreeStar).

### ELISA and ELISPOTS

Antibody secreting cells (ASCs) were assessed using ELISPOTs. Spleen (1 × 10^6^ cells and 0.5 × 10^6^ cells) or bone marrow cells (1 × 10^6^ cells and 0.5 × 10^6^ cells) were added to 96-well cellulose ester membrane plates (MAHAS4510; Millipore) coated with NP_13_-BSA (10 μg/mL) or GPI_8_-BSA (10 μg/mL) and incubated at 37°C and 5% CO_2_ for 8 h. To measure total IgG, wells were coated with sheep anti-mouse IgG (#AC111; Millipore) at 10 μg/mL. Concentration of cells to measure total IgG ASCs were 1 × 10^5^, 0.5 × 10^5^, and 0.1 × 10^5^ per well. Anti-NP IgG, anti-GPI IgG or total IgG ASCs were detected using goat anti-mouse IgG conjugated to ALP (#3310-4; MabTek) and visualized using the BCIP/NBT system (# 50-81-00; KPL, USA). Spots were counted using an automated reader (AID ELISPOT Reader System, software version 7) and calculated as ASCs/1 × 10^6^.

Blood was collected and serum separated at times indicated for ELISA. 96-well MaxiSorp plates were coated with GPI-BSA (10 ng/well) and titrated serum was incubated for at least 24 h at 4°C. Anti-GPI IgG was detected using goat anti-mouse IgG-HRP (SouthernBiotech) and visualized with 3,3′,5,5′- tetramethylbenzidine (TMB) substrate (#T8665, Sigma Aldrich). Antibody concentrations were quantitated using standard curves of known IgG protein concentrations.

### Statistical Analysis

Median values were compared by Mann-Whitney or Wilcoxon matched-pairs signed rank test where appropriate, using Prism version 7 software (GraphPad). Statistical significance comparing more than two groups in an analysis was determined using Kruskal-Wallis followed by Dunn's multiple comparison test as indicated.

## Results

### Synthetic GPI-KLH Vaccination Induced Activation of B Cells Including Class-Switched Antigen-Specific and Memory B Cells

**Figure 1:**
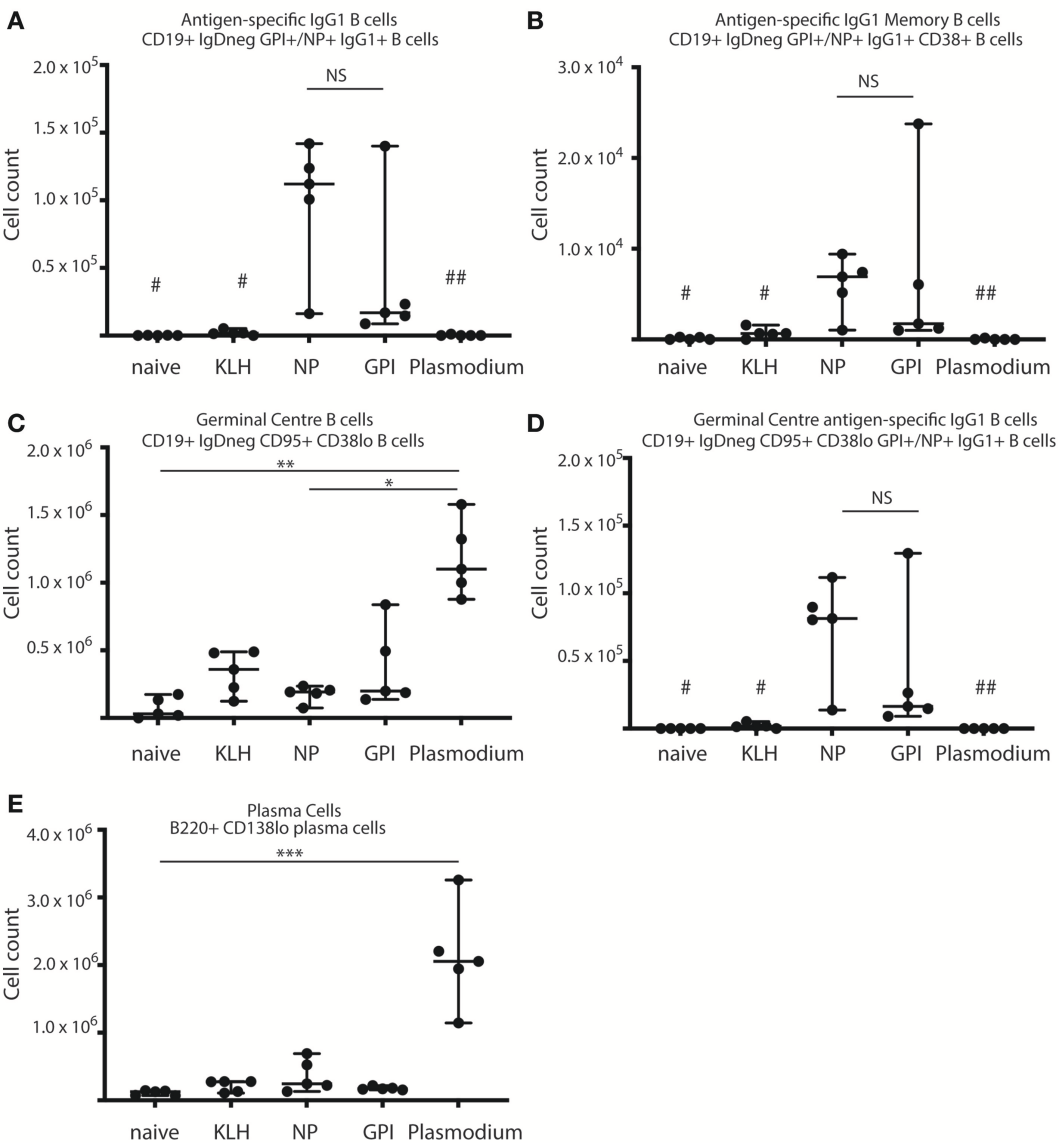
Synthetic GPI-KLH vaccination induced B cell activation. Mice were vaccinated and boosted with NP-KLH (*n* = 5), GPI-KLH (*n* = 5), or KLH alone (*n* = 5) (20 μg/μL) or infected twice with 10^6^ pRBCs then 10^4^ pRBCs (Plasmodium, *n* = 5). Naïve mice (*n* = 5) were included as a baseline. At day 21, activated B cell populations were analyzed by flow cytometry and the number of cells per spleen for **(A)** antigen-specific IgG1 B cells, **(B)** antigen-specific IgG1 memory B cells (MBCs), **(C)** germinal center (GC) B cells, **(D)** antigen-specific IgG1 GC B cells, and **(E)** Plasma cells (PCs) were determined for each mouse. Pooled data from all mice are represented as median ± 95%CI. Data was analyzed using the non-parametric Kruskal-Wallis test and differences determined by Dunn's multiple comparisons test. Significance was determined at *P* < 0.05. NS not significant, ^#^naïve and KLH-immunized mice were significantly different to both groups of NP-KLH and GPI-KLH mice *P* < 0.05, ^##^Plasmodium mice were significantly different to GPI-KLH mice *P* < 0.01, *P < 0.05, **P < 0.01, ***P < 0.001.

Previous studies have shown the importance of antibodies against GPI for protection against severe disease (15, 16) and GPI-KLH vaccination protects against systemic inflammation and fatality in mice (11). However, B cell responses to GPI have not yet been characterized in humans or mice, either to infection or GPI-KLH vaccination, and few studies describe the antigen-specific B cell response to malaria in mouse experimental systems. We sought to assess whether B cells proliferated in response to standard GPI-KLH vaccination or native GPI expressed by *Plasmodium*, and determine which B cell subset populations were involved. Mice were vaccinated twice with NP-KLH, GPI-KLH, or KLH alone according to standard immunization protocols or exposed to pRBC twice (referred to as Plasmodium) (Supplementary Table 1) and euthanized at days 21 or 46. NP-KLH was used as a reference antigen since B cell kinetics has been well-documented (23). Mice vaccinated with KLH alone were included as controls to ensure the flow cytometry probe was GPI-specific and not cross-reacting with KLH-specific B cells. B cells were measured using flow cytometry to quantify subsets of B cells (Figure 1).

B cell activation in response to vaccination with GPI-KLH was similar to NP-KLH at day 21 (Figures 1A–E). This was determined by comparable numbers of cells that had undergone class-switching to IgG1 (Figure 1A), presence of antigen-specific IgG1 MBCs (Figure 1B), number of germinal center (GC) B cells (Figure 1C), antigen-specific IgG1 B cells within the GC (Figure 1D), and the number of plasma cells (PCs) (Figure 1E). Splenocyte counts of activated and antigen-specific IgD^−^GPI^+^IgG1^+^ B cells from GPI-KLH vaccinated mice were significantly higher than the control groups (naïve, KLH) and Plasmodium mice, both overall and within the GC (Figures 1A,D). Furthermore, assessment of IgD^−^GPI^+^IgG1^+^CD38^+^ B cells, whereby CD38^+^ represents murine MBCs (24) showed that these cells were significantly higher in GPI-KLH vaccinated mice compared to naïve, KLH, or the Plasmodium experimental groups. Overall, Plasmodium mice had significantly higher numbers of GC B cells than naïve and NP-KLH mice (Figure 1C), but these were not specific for GPI (Figure 1D). These Plasmodium mice also had a significantly expanded population of PCs at day 21 when compared to naïve mice (Figure 1E). See Supplementary Figure 2 for representative flow plots.

**Figure 2:**
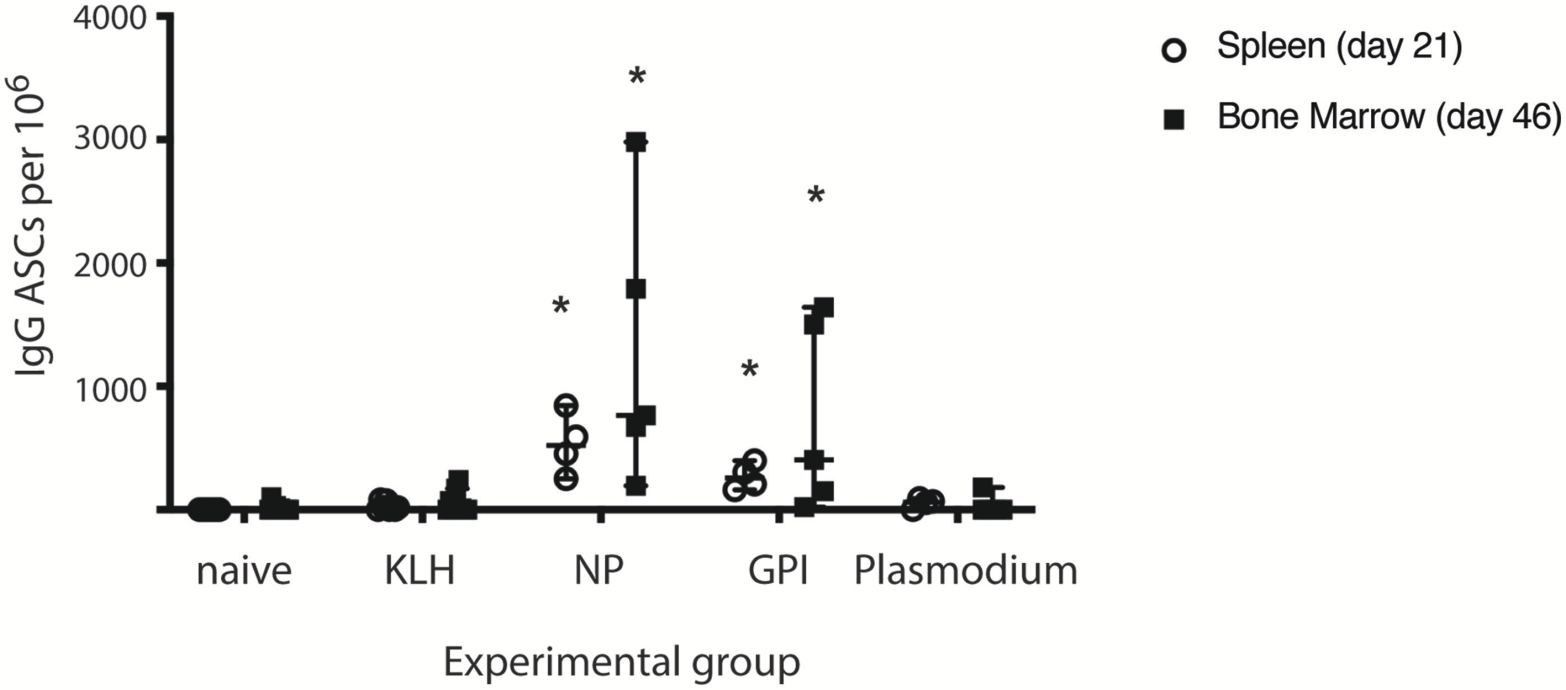
Synthetic GPI-KLH vaccination induced antigen-specific IgG antibody secreting cells (ASCs) in spleen (day 21) and bone marrow (day 46). Mice were vaccinated and boosted with NP-KLH (*n* = 5), GPI-KLH (*n* = 5), or KLH alone (*n* = 5) (20 μg/μL) or infected twice with 10^6^ pRBCs then 10^4^ pRBCs (Plasmodium, *n* = 5). Naïve mice (*n* = 5) were included as a baseline. Splenocytes (open circles, day 21), or lymphocytes (filled squares, day 46) were isolated from the spleen and bone marrow, respectively, and cell counts of antibody secreting cells (ASCs) were quantitated. Pooled data from all mice are represented as median ± 95%-CI. Data was analyzed using the non-parametric Kruskal-Wallis test and differences determined by Dunn's multiple comparisons test. Significance was determined at *P* < 0.05. *Statistically higher than naïve, KLH-vaccinated, or Plasmodium mice at both day 21 and 46. *P* < 0.05.

The capacity of PCs to secrete antigen-specific IgG was measured from isolated splenocytes (day 21) or isolated lymphocytes from bone marrow (Day 46) in all experimental groups (naïve, KLH, NP-KLH, GPI-KLH, and Plasmodium; Figure 2). Significantly higher counts of anti-GPI IgG antibody secreting cells (ASCs) were observed from the spleens and bone marrow of GPI-KLH vaccinated mice than Plasmodium or control mice (naïve, KLH) which was similar in numbers to anti-NP IgG ASCs in the NP-KLH vaccinated mice (Figure 2).

### Challenge With Synthetic GPI-KLH or pRBCs Induced Rapid Activation of GPI-Specific MBCs in Previously GPI-Vaccinated Mice

**Figure 3:**
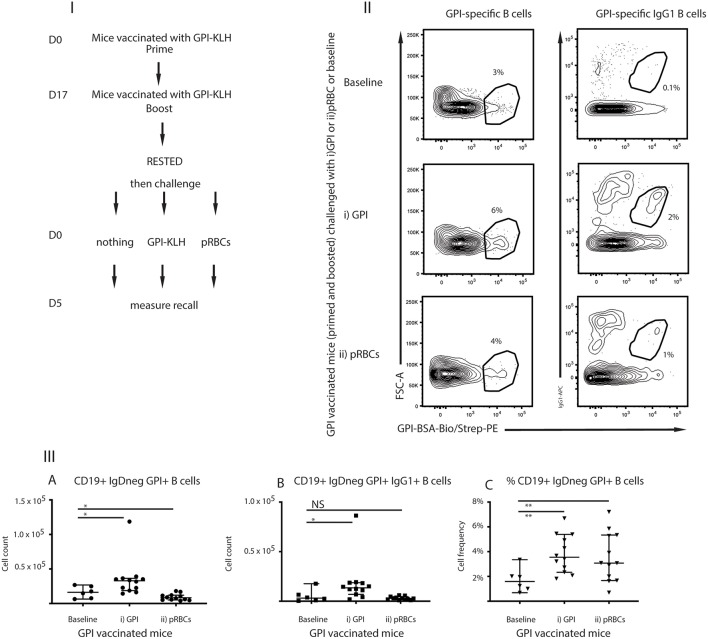
Challenge with (i) synthetic GPI-KLH or (ii) pRBCs induced rapid recall/activation of GPI-specific MBCs in previously GPI-vaccinated mice compared to baseline memory mice. Panel **(I)** Mice were vaccinated and boosted with GPI-KLH (*n* = 30). After resting for a minimum of 10 weeks when the B cells are considered to be in the quiescent memory phase, mice were injected with (i) GPI-KLH *i.p*. (*n* = 12) or (ii) 1 × 10^5^ pRBCs *i.p*. (*n* = 12) or nothing (baseline; *n* = 6) to assess reactivation of MBCs. Panel **(II)** outlines FACS plots. Panel **(III)** is the analysis of B cell populations for the number of cells per spleen for (A) GPI-specific B cells, (B) GPI-specific IgG1 B cells, and (C) calculated cell frequencies of GPI-specific B cells as a proportion of all activated B cells. Experiments were reproduced independently thrice. Pooled data from all mice from all 3 experiments are represented as median ± 95%-CI. Data was analyzed using the non-parametric Mann-Whitney test and significance was determined at *P* < 0.05. *P < 0.05, **P < 0.01, NS not significant.

For GPI to be an effective vaccine target, GPI-specific MBCs must be functional to facilitate long-term immunity. Functionality can be characterized by the ability to rapidly expand upon antigenic challenge as measured by antigen-specific B cells and production of ASCs (25). To investigate recall of GPI-specific MBCs in response to antigenic challenge, mice that had been vaccinated with GPI-KLH were rested for 10 weeks following vaccination to enter the quiescent memory phase (1). Mice were either left unchallenged (referred to as baseline) or challenged with (i) synthetic GPI-KLH or (ii) pRBCs (Figure 3I). Five days later splenocytes were isolated to assess antigen specific B cells using flow cytometry (Figures 3II,III).

Absolute numbers of GPI-specific B cells reactivated significantly in mice following challenge with synthetic GPI-KLH, compared to baseline mice at day 5 (Figures 3IIIA–C). Non-specific depression of proliferation of lymphocytes by malaria infection has been previously noted (26, 27) and therefore, as expected, *P. berghei* infection induced cellular depression (Figures 3IIIA,B). Cellular frequencies in this instance were an indication of cellular reactivation in response to infection as there was a significant increase in GPI-specific B cells (ratio of all activated B cells) when compared to baseline (Figure 3IIIC).

**Figure 4:**
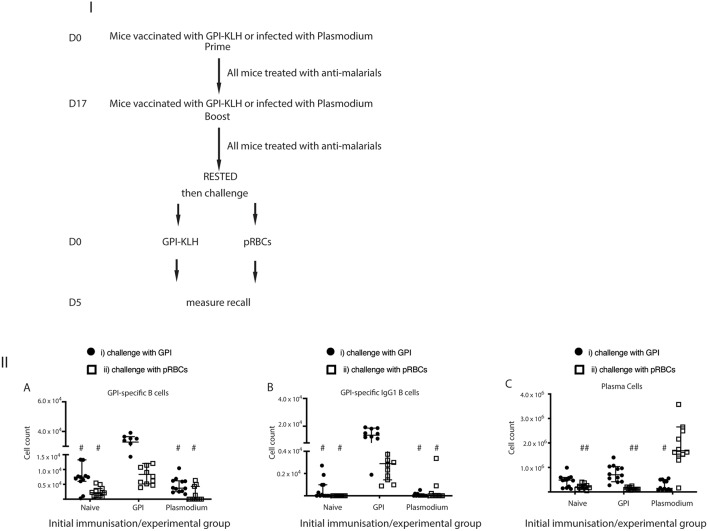
Challenge with synthetic GPI-KLH or pRBCs induced rapid activation of GPI-specific MBCs in previously GPI-vaccinated mice, whilst Plasmodium mice do not generate GPI-specific MBC. Mice were vaccinated and boosted with GPI-KLH (*n* = 24) or infected twice with pRBCs *i.p*. (*n* = 24, referred to as Plasmodium mice). Naïve mice were included as a control group (*n* = 24). After resting for a minimum of 10 weeks, mice were injected with GPI-KLH *i.p*. (closed circles; *n* = 12 from each group) or 1 × 10^5^ pRBCs *i.p*. (open squares; *n* = 12 from each group) to assess reactivation of MBCs at day 5. Panel **(I)** outlines experimental design. Panel **(II)** is the analysis of activated B cell populations for the number of cells per spleen for (A) GPI-specific B cells, (B) GPI-specific IgG1 B cells, and, (C) Plasma Cells at day 5. Experiments were reproduced independently thrice. Pooled data from all mice from all 3 experiments are represented as median ± 95%-CI. Data was analyzed using the non-parametric Kruskal-Wallis test and differences determined by Dunn's multiple comparisons test. Significance was determined at *P* < 0.05. (A,B) ^#^when challenged with GPI-KLH or pRBCs, the initial experimental groups of naïve mice and Plasmodium mice were significantly lower than GPI-KLH mice (*P* < 0.05). (C) ^#^when challenged with GPI-KLH the initial experimental group of Plasmodium mice was significantly lower than the GPI-KLH mice (*P* < 0.05); ^*##*^when challenged with pRBCs, the initial experimental groups of naïve and GPI-KLH mice were significantly lower than Plasmodium mice for plasma cell production (*P* < 0.05).

To establish whether these GPI specific B cells were in fact originating from reactivation of MBCs rather than activation of naïve B cells against foreign antigen, we also challenged naïve mice with synthetic GPI-KLH or pRBCs and quantified GPI-specific B cells (Figure 4; Supplementary Figure 3). When challenged with synthetic GPI-KLH or pRBCs, there was no evidence of GPI specific B cells or GPI^+^IgG1^+^ B cells in naïve mice (Figures 4IIA,B). When challenged with synthetic GPI-KLH, PC production in the spleen was lower in naïve mice than GPI-KLH vaccinated mice, but this difference was not statistically significant (Figure 4IIC). Similarly, when infected with pRBCs, PC counts from naïve mice were not different from GPI-KLH vaccinated mice (Figure 4IIC).

Since we showed that infection with pRBCs, and thus parasite GPI, induced GPI specific MBC activation in previously vaccinated mice compared to baseline (Figure 3IIIC) and naïve mice (Figures 4IIA,B) we sought to investigate if Plasmodium mice underwent GPI-specific MBC reactivation when challenged with synthetic GPI-KLH or a third infection with pRBCs (Figures 4IIIA,B). It is conceivable that B cells could have generated GPI-specific memory during the initial infections in the Plasmodium group against native parasite GPI (28). However, we observed that GPI-specific B cell counts, including GPI^+^IgG1^+^ B cells and GPI^+^IgG1^+^ GC B cells, were significantly higher in vaccinated mice when challenged with GPI-KLH or pRBCs than Plasmodium mice (Figures 4IIA,B). There were no significant differences between Plasmodium or naïve mice for antigen specific cell counts. Furthermore, when challenged with GPI-KLH, vaccinated mice generated significantly higher numbers of PCs than Plasmodium mice (Figure 4IIC). On the contrary, when both groups were challenged with pRBCs, only the Plasmodium mice had significantly higher PC counts than both GPI-KLH vaccinated mice and naïve mice (Figure 4IIC). This was further investigated using ELISPOTs.

### MBC Are Functional ASCs in GPI-Vaccinated Mice and Plasmodium Mice When Challenged With GPI-KLH or pRBCs

**Figure 5:**
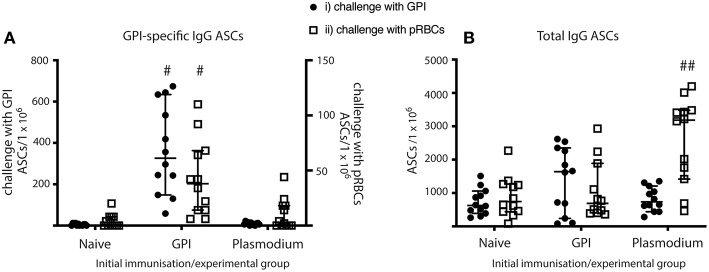
MBC differentiate into ASCs and produce antibodies in vaccinated mice when given GPI-KLH or pRBCs, and in Plasmodium mice when given a third dose of pRBCs. Mice were vaccinated and boosted with GPI-KLH (*n* = 24) or infected twice with pRBCs *i.p*. (*n* = 24, referred to as Plasmodium mice). Naïve mice were included as a control group (*n* = 24). After resting for a minimum of 10 weeks, mice were injected with GPI-KLH *i.p*. (*n* = 12 from each group) or 1 × 10^5^ pRBCs *i.p*. (*n* = 12 from each group) to assess reactivation of MBCs and differentiation into ASCs at day 5 using ELISPOTs. **(A)** Denotes the proportion of GPI-specific ASCs/1 × 10^6^ cells for challenge with synthetic GPI (closed circle) or pRBCs (open square). **(B)** Denotes the proportion of total ASCs/1 × 10^6^ cells for challenge with synthetic GPI (closed circle) or pRBCs (open square). Pooled data from all experimental mice are represented as median ± 95%-CI. Data was analyzed using the non-parametric Kruskal-Wallis test and differences determined by Dunn's multiple comparisons test. Significance was determined at *P* < 0.05. ^#^GPI-specific ASCs in spleens of GPI-vaccinated mice statistically higher than naïve and Plasmodium mice when challenged with both GPI or pRBCs (*P* < 0.05); ^##^total IgG in spleens of Plasmodium mice statistically higher than naïve and GPI mice when challenged with pRBCs (*P* < 0.01).

MBC functionality can be measured by rapid differentiation into ASCs. From the flow cytometry analysis, we observed high frequencies of PCs in GPI-KLH vaccinated mice when challenged with GPI-KLH, but these counts were only significant compared to Plasmodium mice (Figure 4IIC). Additionally, when challenged with pRBCs, Plasmodium mice had a considerable population of PCs which was significantly higher than both GPI-KLH vaccinated and naïve mice (Figure 4IIC). From these observations, we sought to determine in each experimental group if spleen PCs were secreting the isotype IgG and whether these were GPI-specific (Figure 5). To this end, we used ELISPOTS coated with GPI-BSA or sheep anti-mouse total IgG to detect anti-GPI IgG ASCs or total IgG ASCs, respectively.

GPI-vaccinated mice had significantly higher counts for anti-GPI IgG ASCs than both Plasmodium and naïve mice when challenged with either GPI-KLH or pRBCs (Figure 5A). Plasmodium mice had significantly higher total IgG ASCs than both naïve and GPI-KLH vaccinated mice when challenged with a third dose of pRBCs (Figure 5B).

**Figure 6:**
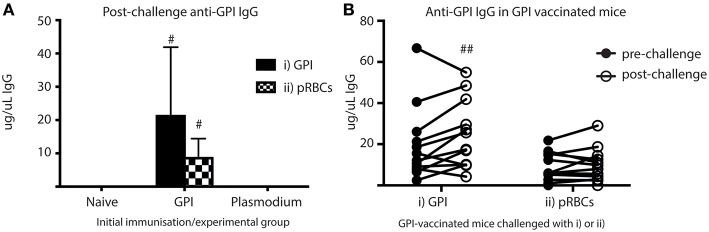
Higher titers of anti-GPI IgG at day 5 in GPI-KLH mice following injection with synthetic GPI-KLH or pRBC compared to naïve or Plasmodium mice. Mice were vaccinated and boosted with GPI-KLH (*n* = 24) or infected twice with pRBCs *i.p*. (*n* = 24). Naïve mice were included as a control (*n* = 24). Mice were rested for 10 weeks. Mice were subsequently challenged with GPI-KLH *i.p*. or 1 × 10^5^ pRBCs *i.p*. and post-challenge sera collected from a cardiac bleed at day 5. **(A)** Day 5 titers were compared among groups to measure titers of anti-GPI IgG in response to GPI-KLH (filled bars; *n* = 12 from each group) or pRBCs (checked bars; *n* = 12 from each group). Pooled data from all experimental mice was analyzed using the non-parametric Kruskal-Wallis test and differences determined by Dunn's multiple comparisons test. Significance was determined at *P* < 0.05. **(B)** In the GPI-KLH vaccinated group, sera samples were taken up to 7 days prior to challenge with GPI-KLH or pRBCs and titers measured (closed circles; pre-challenge). These titers were pair-matched to day 5 post-challenge titers (open circles; post-challenge) using the Paired Wilcoxon test to measure potential boosting of anti-GPI IgG in response to GPI-KLH or pRBCs. Significance was determined at *P* < 0.05. Data are represented as median ± 95%-CI. ^#^GPI-KLH vaccinated mice statistically higher than naïve mice and Plasmodium mice (*P* < 0.001). ^##^significant increase in anti-GPI IgG comparing pre- and post-challenge sera titers (*P* < 0.05).

To investigate if ASCs also secreted significant quantities of anti-GPI IgG into the blood *in vivo*, sera samples taken from cardiac bleeds at day 5 (post-challenge) were analyzed for anti-GPI IgG using ELISAs (Figure 6A). Anti-GPI IgG measured in the sera of GPI-KLH vaccinated mice was significantly higher compared to naïve and Plasmodium mice after challenge with either GPI-KLH or pRBCs (Figure 6A). To determine if this was a boosting effect in response to challenge, or residual high levels induced from earlier vaccination, sera samples from GPI-KLH vaccinated mice were taken up to a week prior to challenge (pre-challenge) and compared to the post-challenge titer using paired Wilcoxon tests (Figure 6B). Synthetic GPI-KLH challenge produced a significant boosting of anti-GPI IgG titers and, although pRBC challenge also boosted titers of anti-GPI IgG, this was not significant (Figure 6B). Little detectable anti-GPI IgG was measured for naïve or Plasmodium mice.

## Discussion

Activation of antigen-specific B cells in response to foreign antigen, and the subsequent affinity maturation, class-switching, MBC production, long-lived PCs and antibody production play an integral role in adaptive humoral immunity (29). Carbohydrate antigens are weakly immunogenic and classified as Thymus-Independent (TI) type I or II (30). In order to generate the above favorable Thymus Dependent (TD) response, carbohydrates can be manipulated in different vaccine regimes by the use of adjuvants or conjugation to other immunodominant protein carriers. Historically carbohydrate-conjugated vaccines have been successful for reducing incidence of diseases and the advancement in glycobiology has allowed carbohydrate-based vaccines to gain momentum for their potentially broad application in other infectious diseases including malaria (11, 31). Our study has for the first time been able to detect carbohydrate-malaria-specific B cells and the subsequent reactivation and boosting of these functional MBCs in response to both synthetic carbohydrate antigen and *Plasmodium* blood-stage infection. Importantly, this was achieved through developing a GPI-BSA-BIO probe, which enabled phenotypic assessment of activated carbohydrate-specific B cells following vaccination and re-activation in response to infection with minimal *in vitro* manipulation.

TI-I and II responses differ via their mechanism of B cell activation. TI-I antigens are further classified based on antigen concentration. In high concentrations, foreign antigen binding is via Toll-Like Receptors (TLRs) inducing mitogenic non-specific polyclonal activation of B cells, thereby precluding Ig epitope recognition and are often deleterious to the host (30). As the concentration of these antigens reduce, cross-linking of the Ig can occur via epitope recognition enabling antigen-specific activation of B cells and formation of functional MBCs. However, there MBCs are predominantly short-lived IgM MBCs (32). A well-characterized TI-I antigen is LPS and different doses activate B cells via these different pathways (33). Since GPI behaves like LPS, it is feasible that the biological mechanism of natural infection with native GPI would also be similar. Therefore, the proposed vaccine construct GPI-KLH has been conjugated to a carrier protein to favor TD responses and generation of class-switched functional MBCs.

The hapten NP has successfully been used to track antigen-specific B cell responses. As such, we employed the same NP-KLH system as a means to compare the responses of our GPI-KLH vaccine. We found that B cells were activated in response to GPI-KLH similarly to the standard hapten NP-KLH. Vaccination elicited class-switching, antigen specificity, long-lived PCs, sera titers of anti-GPI IgG, and IgG1^+^GPI^+^ MBCs. This is indicative of a TD response and favorable characteristics of humoral-based vaccines (29). However, in addition to eliciting an appropriate response, the reactivation of antigen-specific MBCs upon subsequent antigenic challenge is the ultimate goal for clinicians. This is especially important in light of poor immunological responsiveness to RTS,S.

We used a similar protocol to Krishnamurty et al. (1) to show that GPI-specific MBCs generated in response to GPI-KLH vaccination were reactivated upon synthetic GPI-KLH challenge or infection with *Plasmodium* (pRBCs). Synthetic GPI-KLH induced significant cellular increases in GPI-specific B cells, anti-GPI IgG ASCs, and boosting of sera titers. This response was indicative of memory recall, since naïve mice did not have a detectable antigen-specific response at this time. Boosting and reactivation of MBCs agrees with other carbohydrate immunizations (34, 35).

When challenged with *P. berghei*, there was a significantly higher % of GPI-specific B cells complementing a significant generation of anti-GPI secreting PCs in the spleen. Furthermore, when these infected vaccinated mice were compared to infected naïve mice or previously infected mice (Plasmodium mice) challenged with pRBCs for the third time, there was a significantly higher post-challenge sera titer of anti-GPI IgG. Although it is not possible to conclude from this study what underlying cellular processes involved in this recall response in vaccinated mice with native GPI are, it could be speculated that native GPI during infection is binding via epitope recognition on surface Ig on the functional MBCs that were formed during initial vaccination, thereby allowing their controlled expansion and boosting. The mechanisms behind this recall activation and how this differs from reactivation using synthetic GPI-KLH challenge remains to be defined. Nevertheless, these observations have significant clinical implications creating a system whereby natural infection will continue to boost and clonally expand MBCs in vaccinated mice.

In contrast, MBC reactivation in previously infected mice challenged with either synthetic GPI-KLH or a third infection did not induce GPI-specific responses. In the previous infections in these Plasmodium mice, the higher concentrations of native GPI has likely inhibited the antigen-specific activation of B cells through mitogenic polyclonal TLR activation. Therefore, it is not surprising that we cannot detect GPI-specific B cells in response to challenge with either synthetic GPI nor a third infection with native GPI. Another explanation could be activation of the enzyme indoleamine 2,3 dioxygenase which occurs during malaria infection (27). This enzyme is important for tryptophan metabolism and has been shown to inhibit B cell responses to TI antigens (26). Despite which mechanism is responsible, these findings agree with Keitany et al. (8) who also found that antigenic challenge in previously infected mice failed to induce antigen-specific MBC reactivation.

We have shown for the first time that GPI-specific MBC recall can be observed in vaccinated mice in response to infection with *Plasmodium*. The extent to which findings in the experimental mouse model should be extrapolated to human disease should be viewed with caution. Future studies need to establish whether affinity matured, isotype switched MBCs can be generated during a GPI-KLH vaccine regime administered to a previously *Plasmodium* infected mouse in the quiescent phase when parasites are cleared. This would ensure that functional MBCs can be generated after previous infection, which would mimic vaccination in endemic regions. Future studies may also investigate the kinetic response of GPI B cell reactivation following pRBC challenge at later time points in order to track secondary GC formation following antigen re-exposure, as well as the protective efficacy of vaccination.

We have also established that natural infection with *Plasmodium* is unlikely to generate efficient GPI-specific MBCs, potentially because of the mechanism by which GPI drives activation and differentiation of B cells as a TI-I antigen. By utilizing the glycoconjugate in a vaccine formulation, and altering the mechanism of antigen presentation, vaccinated mice do generate functional GPI-specific MBCs capable of recall and boosting upon natural infection. Our research raises interesting biological questions and observations about the potential mechanisms by which *Plasmodium* drives differentiation and activation of B cells, and the phenomenon of inefficient or partial immunity as a result of this, through use of different activation signaling pathways within naïve B cells. It has become essential to understand and learn the mechanisms contributing to MBC failure in natural infection in order to appropriately target next generational malaria vaccines. Our research has significant clinical implications for including glycan targets in the malaria elimination agenda, either as a complement to RTS,S or when pursuing new vaccine formulations.

## Ethics Statement

For all experiments 6- to 8-week old inbred male and female C57BL/6 mice were used and all procedures complied with the Walter and Eliza Hall Institute Animal Ethics Committee requirements.

## Author Contributions

LS and EE provided the conceptual framework for the study. HJ and LS designed the experiments. HJ developed the probe, performed all experiments, analyzed the data, and wrote the manuscript. QT and RM provided the conjugated vaccine. EE provided critical support for flow cytometry data analysis. LS and EE critically reviewed the manuscript.

### Conflict of Interest Statement

The authors declare that the research was conducted in the absence of any commercial or financial relationships that could be construed as a potential conflict of interest.

## Supplementary Material

See Supplementary Table 1 for infection and immunization schedules. See Supplementary Figure 1 for the flow cytometry gating strategy including representative plots for FMOs and BSA-biotin background staining. See Supplementary Figure 2 for representative FACS plots of experimental groups. See Supplementary Figure 3 for a representative FACS plots for the recall experiment.

Supplementary Figure 1 Flow cytometry gating strategy. This is the flow cytometry gating strategy using an example of a spleen isolated from a GPI-immunized mouse at day 14.

Supplementary Figure 2 Representative plots for experimental groups; one mouse from each group.

Supplementary Figure 3 Challenge with synthetic GPI-KLH or pRBCs induced rapid activation of GPI-specific MBCs in previously GPI-vaccinated mice, whilst Plasmodium mice do not generate GPI-specific MBC. Mice were vaccinated and boosted with GPI-KLH (*n* = 24) or infected twice with pRBCs *i.p*. (*n* = 24, referred to as Plasmodium mice). Naïve mice were included as a control group (*n* = 24). After resting for a minimum of 10 weeks, mice were injected with GPI-KLH *i.p*. or 1 × 10^5^ pRBCs *i.p*. to assess reactivation of MBCs at day 5. The supplementary figure outlines FACS plots.

Supplementary Table 1 Vaccination/Infection protocol for assessing B cell activation kinetics and memory B cell recall.

## Abbreviations

BIO: biotin labelling
BSA: bovine serum albumin
GPI: glycosylphosphatidylinositol
KLH: Keyhole Limpet Haemocyanin
MBC: memory B cells
NP: Four-hydroxy-3-nitrophenyl acetyl-Osu
pRBCs: parasitized red blood cells.
