# Supplementary figures and images for: Vaccine-Induced Carbohydrate-Specific Memory B Cells Reactivate During Rodent Malaria Infection
Source: Front Immunol. 2019 Aug 9;10:1840. doi: 10.3389/fimmu.2019.01840 (PMC6696980; doi:10.3389/fimmu.2019.01840)

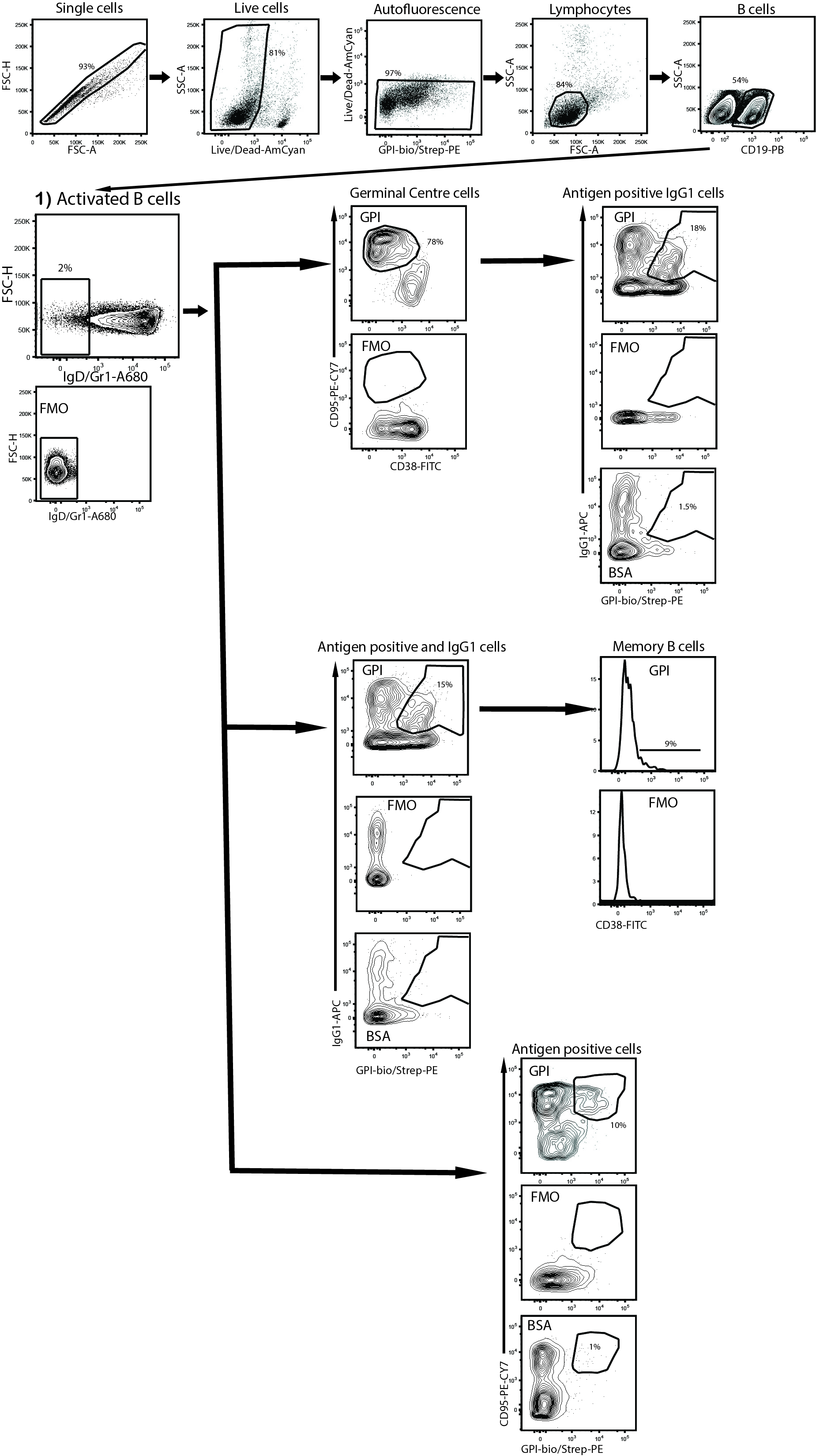

Supplement: Supplementary Figure 1 — Flow cytometry gating strategy. This is the flow cytometry gating strategy using an example of a spleen isolated from a GPI-immunized mouse at day 14. [file Image_1.TIF]

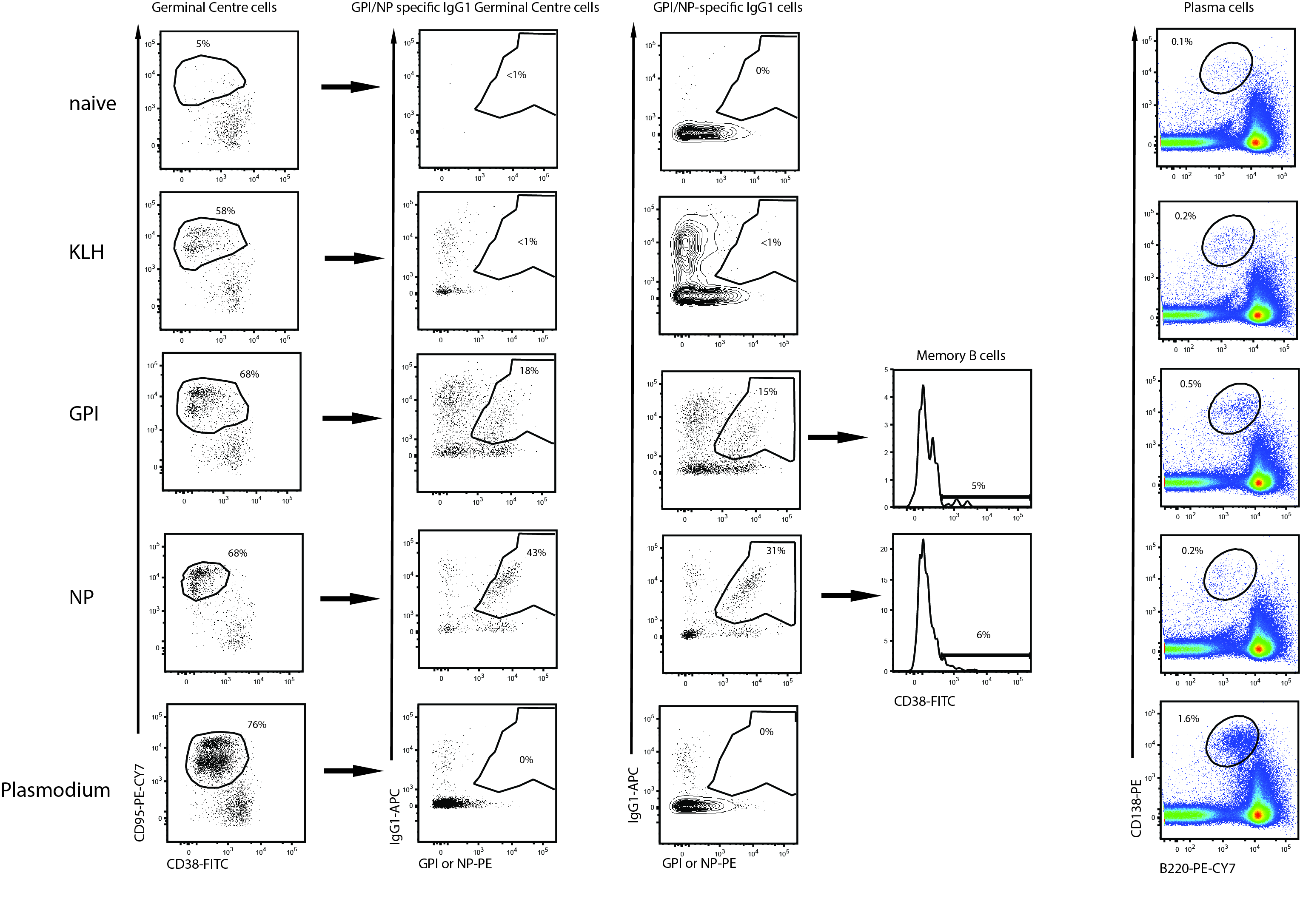

Supplement: Supplementary Figure 2 — Representative plots for experimental groups; one mouse from each group. [file Image_2.TIF]

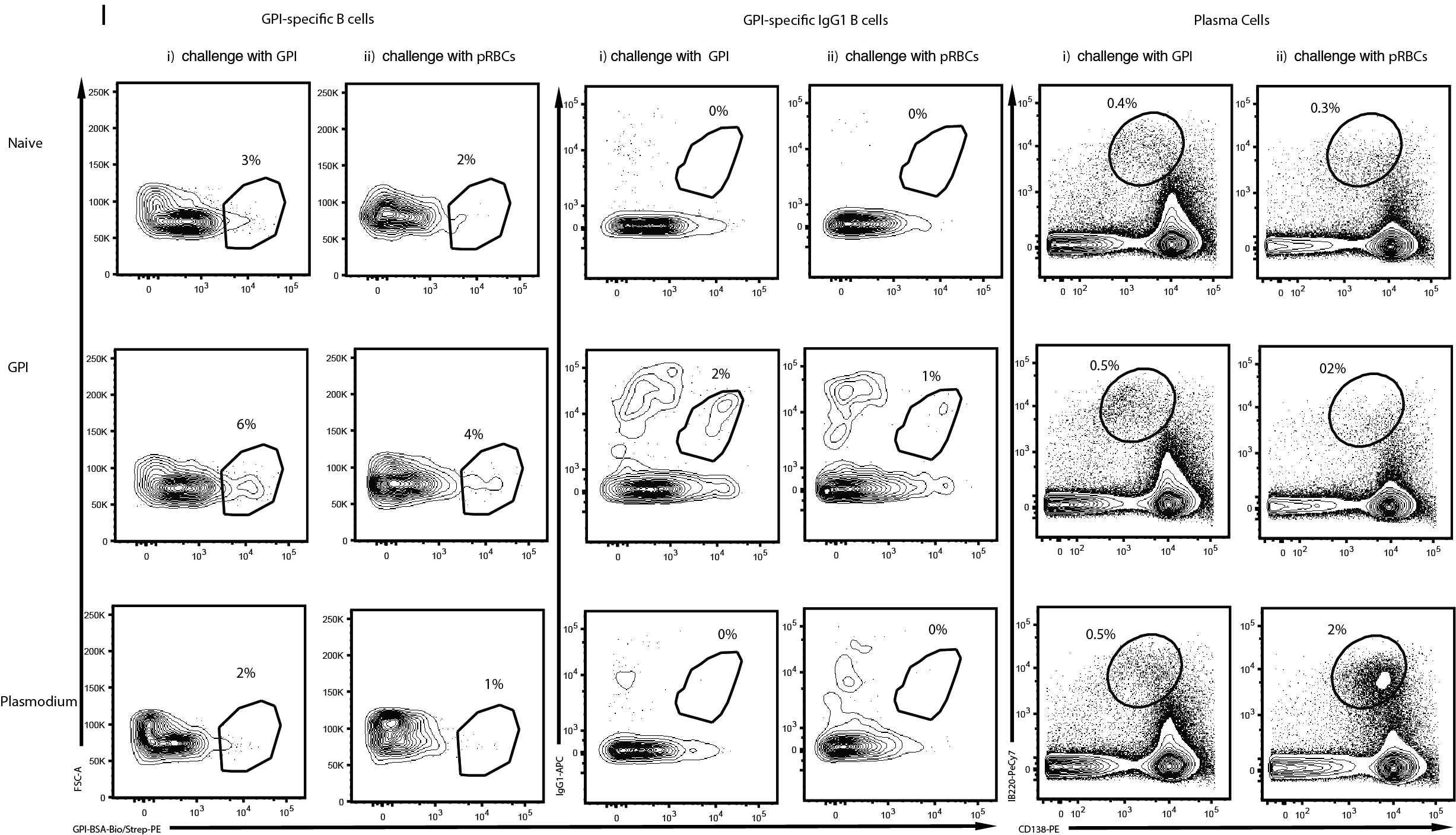

Supplement: Supplementary Figure 3 — Challenge with synthetic GPI-KLH or pRBCs induced rapid activation of GPI-specific MBCs in previously GPI-vaccinated mice, whilst Plasmodium mice do not generate GPI-specific MBC. Mice were vaccinated and boosted with GPI-KLH (n = 24) or infected twice with pRBCs i.p. (n = 24, referred to as Plasmodium mice). Naïve mice were included as a control group (n = 24). After resting for a minimum of 10 weeks, mice were injected with GPI-KLH i.p. or 1 × 10^5^ pRBCs i.p. to assess reactivation of MBCs at day 5. The supplementary figure outlines FACS plots. [file Image_3.TIF]
