# Supplementary material for: Vaccine-Induced Carbohydrate-Specific Memory B Cells Reactivate During Rodent Malaria Infection
Source: Front Immunol. 2019 Aug 9;10:1840. doi: 10.3389/fimmu.2019.01840 (PMC6696980; doi:10.3389/fimmu.2019.01840)
Supplement: Supplementary Table 1 — Vaccination/Infection protocol for assessing B cell activation kinetics and memory B cell recall. [file Table_1.DOCX]

**Supplementary Table 1: Vaccination/Infection protocol for assessing B cell activation kinetics and memory B cell recall.**

|  | **Immune activation of B cells** | | | | | | **MBC recall** | | |
| --- | --- | --- | --- | --- | --- | --- | --- | --- | --- |
| **Group** | **Day 0** | **Day 5** | **Day 17** | **Day 20** | **Day 21** | **Day 46** | **Group** | **Day 90+** | **Day 5** |
| GPI-KLH | 20ug/100uL GPI-KLH *i.p.*  n = 40 | Drug cure | 20ug/100uL GPI-KLH *i.p.* | Drug cure | Flow cytometry and ELISPOT (spleen)  n = 25 | ELISPOT (bone marrow)  n = 25 | GPI-KLH | Nothing n=6 | Flow cytometry and ELISPOT (spleens)  ELISA (sera)  n=78 |
|  |  |  |  |  |  |  |  | 20ug/100uL GPI-KLH^b^ *i.p.* n=12 |  |
|  |  |  |  |  |  |  |  | 10^5^ pRBC^a^ n=12 |  |
| NP-KLH | 20ug/100uL NP-KLH *i.p.*  n = 10 | Drug cure | 20ug/100uL NP-KLH *i.p.* | Drug cure |  |  | Naive | 20ug/100uL GPI-KLH^b^ *i.p.* n=12 |  |
|  |  |  |  |  |  |  |  | 10^5^ pRBC^a^ n=12 |  |
| KLH | 20ug/100uL KLH *i.p.*  n = 10 | Drug cure | 20ug/100uL KLH *i.p.* | Drug cure |  |  | Plasmodium | 20ug/100uL GPI-KLH^b^ *i.p.* n=12 |  |
| Naïve | Nothing  n = 34 | Drug cure | Nothing | Drug cure |  |  |  | 10^5^ pRBC^a^ n=12 |  |
| Plasmodium | 10^6^ pRBC^a^  n = 34 | Drug cure | 10^4^ pRBC^a^ | Drug cure |  |  |  |  |  |

^a^parasitized RBCs

^b^no adjuvant
